# Global methylation, oxidative stress, and relative telomere length in biliary atresia patients

**DOI:** 10.1038/srep26969

**Published:** 2016-05-31

**Authors:** Wanvisa Udomsinprasert, Nakarin Kitkumthorn, Apiwat Mutirangura, Voranush Chongsrisawat, Yong Poovorawan, Sittisak Honsawek

**Affiliations:** 1 Department of Biochemistry, Faculty of Medicine, Chulalongkorn University, King Chulalongkorn Memorial Hospital, Thai Red Cross Society, Bangkok, Thailand; 2 Department of Oral and Biology, Faculty of Dentistry, Mahidol University, Bangkok, Thailand; 3 Center of Excellence in Molecular Genetics of Cancer and Human Diseases, Department of Anatomy, Faculty of Medicine, Chulalongkorn University, Bangkok, Thailand; 4 Center of Excellence in Clinical Virology, Department of Pediatrics, Faculty of Medicine, Chulalongkorn University, King Chulalongkorn Memorial Hospital, Bangkok, Thailand

## Abstract

Alu and LINE-1 elements are retrotransposons with a ubiquitous presence in the human genome that can cause genomic instability, specifically relating to telomere length. Genotoxic agents may induce methylation of retrotransposons, in addition to oxidative DNA damage in the form of 8-hydroxy-2′-deoxyguanosine (8-OHdG). Methylation of retrotransposons induced by these agents may contribute to biliary atresia (BA) etiology. Here, we investigated correlations between global methylation, 8-OHdG, and relative telomere length, as well as reporting on Alu and LINE-1 hypomethylation in BA patients. Alu and LINE-1 hypomethylation were found to be associated with elevated risk of BA (OR = 4.07; 95% CI: 2.27–7.32; *P* < 0.0001 and OR = 3.51; 95% CI: 1.87–6.59; *P* < 0.0001, respectively). Furthermore, LINE-1 methylation was associated with liver stiffness in BA patients (β coefficient = −0.17; 95% CI: −0.24 to −0.10; *P* < 0.0001). Stratified analysis revealed negative correlations between Alu and LINE-1 methylation and 8-OHdG in BA patients (*P* < 0.0001). In contrast, positive relationships were identified between Alu and LINE-1 methylation and relative telomere length in BA patients (*P* < 0.0001). These findings suggest that retrotransposon hypomethylation is associated with plasma 8-OHdG and telomere length in BA patients.

Biliary atresia (BA) is one of the most common causes of neonatal cholestatic liver disease. BA is characterized by a progressive idiopathic fibrosclerotic cholangiopathy that results in obliteration of the extrahepatic biliary tree. Although effective bile flow can be established by Kasai portoenterostomy, the majority of BA patients will ultimately develop severe cholestasis, liver cirrhosis, and end-stage liver disease^1^. The etiologies of BA have not been well established. However, several theories have been proposed to explain the pathogenesis of BA, including viral infections, toxins, and immunologic insults; notably, the interplay between environmental and genetic factors^2^. Growing evidence suggests that epigenetic variation can be elicited by viruses, toxins, and genetic defects^3^, which may have relevance in the development of BA.

DNA methylation, one type of epigenetic change, is a reversible modification of cytosine residues in the genome through the addition of a methyl group to cytosine nucleotides. This variation is an important mechanism in regulating expression of human genes, maintenance of genomic stability, and telomere length^4^. A substantial portion of methylation sites throughout the human genome are found in repetitive sequences and transposable elements, such as Alu or short interspersed nuclear element (SINE) and long interspersed nuclear element-1 (LINE-1). Alu and LINE-1 are major components of non-long terminal repeat retrotransposons, comprising approximately 11% and 17% of the human genome, respectively^5^. Because repetitive DNA sequences account for over 40% of methylation in the genome, DNA methylation measured in retrotransposon elements has served as a useful proxy for global DNA methylation^6^. Alu and LINE-1 elements are usually heavily methylated in normal cells, thus maintaining transcriptional inactivation and inhibiting retrotransposition. Hypomethylation of these elements is hypothesized to facilitate genomic instability by resulting in retrotransposition of transposable elements, dysregulation of DNA repair genes^7^,^8^, and altered expression of important genes^9^. Previous studies have highlighted relationships between global hypomethylation and several human diseases^10^,^11^,^12^,^13^. Methylation of these elements also makes them susceptible to oxidative stress^14^, which may be a possible factor associated with biliary atresia.

Oxidative stress constitutes the majority of DNA damage in human cells, which is due mainly to excess production of reactive oxygen species (ROS)^15^. Generation of ROS can lead to a wide range of DNA lesions, including base deletions, mutations, DNA strand breakage, chromosomal rearrangements, and cross-linking with proteins^16^. Oxidative DNA damage can modify epigenetic alterations by multiple mechanisms. One form of DNA damage induced by oxidative stress is the change in genomic base to species like 8-hydroxy-2′-deoxyguanosine (8-OHdG). 8-OHdG is able to interfere with the ability of DNA to function as a substrate for the DNA methyltransferases (DNMTs), leading to global DNA hypomethylation and subsequent genomic instability^17^. Alu and LINE-1 may be critical elements in chromosome and genomic stability and may be induced by an increase in oxidative stress, leading to genomic instability and DNA damage. As such, these elements may contribute to the pathophysiology of BA. To date, there has been no evidence regarding the possible association between global methylation and oxidative DNA damage in BA patients. This information could improve our understanding of the relationship between epigenetic alteration-mediated DNA damage and BA etiology. Interestingly, epigenetic mechanism appears to be an important component of telomere regulation. Several studies have reported that hypomethylation of subtelomeric regions was related to telomere length and that these regions might be important to epigenetic regulation in telomere maintenance^18^,^19^, thereby establishing a possible etiologic link between global DNA methylation and telomere length in BA patients.

While methylation of retrotranposon elements has been investigated in relation to a variety of disorders, little is known about the association of global DNA methylation and the exact patho-etiology of BA. We hypothesize that epigenetic alterations in the form of global DNA methylation, may be associated with outcome parameters and telomere length in BA patients. Accordingly, the primary aim of the present study was to assess methylation levels and patterns of Alu and LINE-1 elements in peripheral blood leukocytes from BA patients and age-matched healthy controls using quantitative combine bisulfite restriction analysis (qCOBRA). We further investigated whether Alu and LINE-1 methylation levels were associated with hepatic dysfunction, oxidative stress, and relative telomere length in BA patients. Additionally, we examined the association between Alu and LINE-1 methylation and risk of BA. Further understanding of global DNA methylation, oxidative damage, and telomere length would shed light on the role of epigenetic aberrations play in the etiology of BA and may ultimately support the development of effective strategies.

## Results

### Characteristics of study subjects

Baseline demographic characteristics of participants in this analysis are listed in Supplementary Table 1. Of 228 participants enrolled in this study, 114 patients were diagnosed with BA (57.89% female and 42.11% male) and 114 were healthy controls (56.14% female and 43.86% male). There were no significant differences in age or gender between BA patients and healthy controls. However, BA patients had significantly higher liver stiffness, AST, and ALT values than controls (*P* < 0.0001).

### Hypomethylation of Alu and LINE-1 elements in biliary atresia

**Figure 1:**
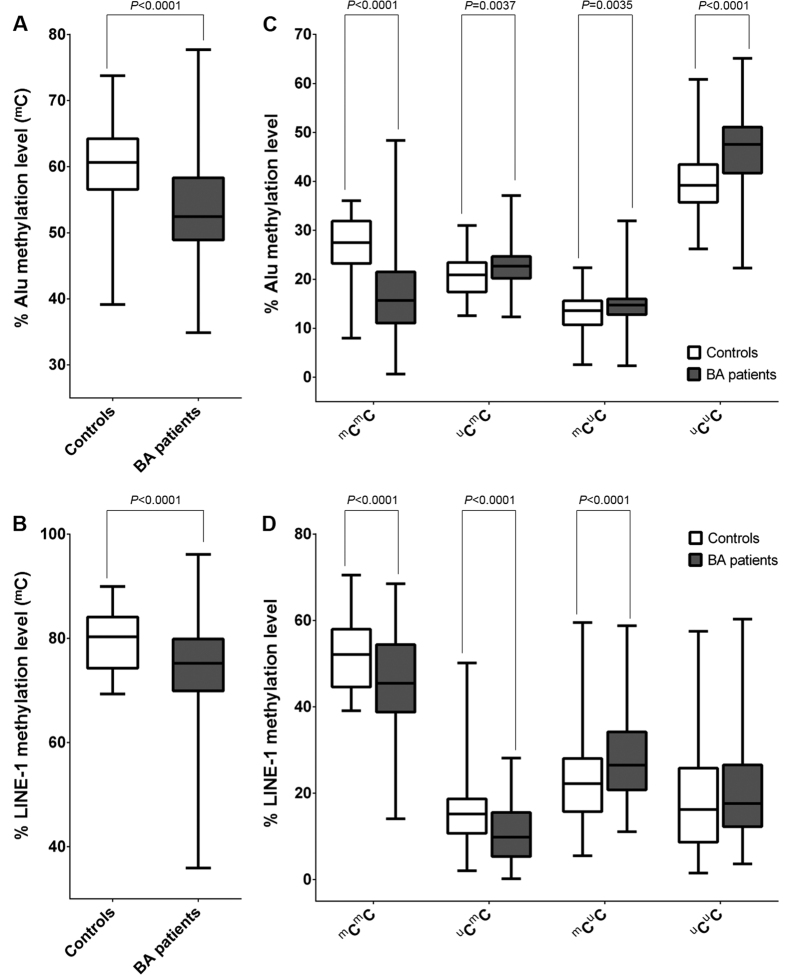
Methylation levels and patterns of Alu and LINE-1 elements in controls and BA patients. (**A**) Alu methylation levels; (**B**) LINE-1 methylation levels; (**C**) Alu methylation patterns; (**D**) LINE-1 methylation patterns.

In order to explore potential epigenetic alterations resulting from global methylation in BA, we measured Alu and LINE-1 methylation in peripheral blood leukocytes of BA patients and age-matched healthy controls. Figure 1A reveals the distribution of Alu methylation levels in BA patients and controls in box plot format. Median Alu methylation level in BA patients was significantly lower than in healthy controls (*P* < 0.0001). LINE-1 methylation levels were also found to be lower in BA patients than in healthy controls (*P* < 0.0001) (Fig. 1B).

We further investigated methylation patterns of Alu and LINE-1 elements in BA patients and healthy controls. Median percentages of each Alu methylation pattern are shown in Fig. 1C. Interestingly, we observed significant elevation of hypomethylation pattern (^u^C^u^C) at Alu elements in BA patients, as compared to healthy controls (*P* < 0.0001). Similarly, BA patients demonstrated higher methylation of partial methylation patterns (^u^C^m^C and ^m^C^u^C) than the control group (*P* = 0.0037 and *P* = 0.0035, respectively). In contrast, the percentage of hypermethylation pattern (^m^C^m^C) was significantly decreased in BA patients (*P* < 0.0001). BA patients had significantly reduced LINE-1 methylation of both hypermethylation pattern (^m^C^m^C) and partial methylation pattern (^u^C^m^C), as compared to controls (*P* < 0.0001 and *P* < 0.0001, respectively) (Fig. 1D). However, the percentage of partial methylation pattern (^m^C^u^C) at LINE-1 elements was significantly higher in BA patients than in unaffected controls (*P* < 0.0001). This was not observed in LINE-1 methylation of hypomethylation pattern in a comparison between cases and controls.

**Figure 2:**
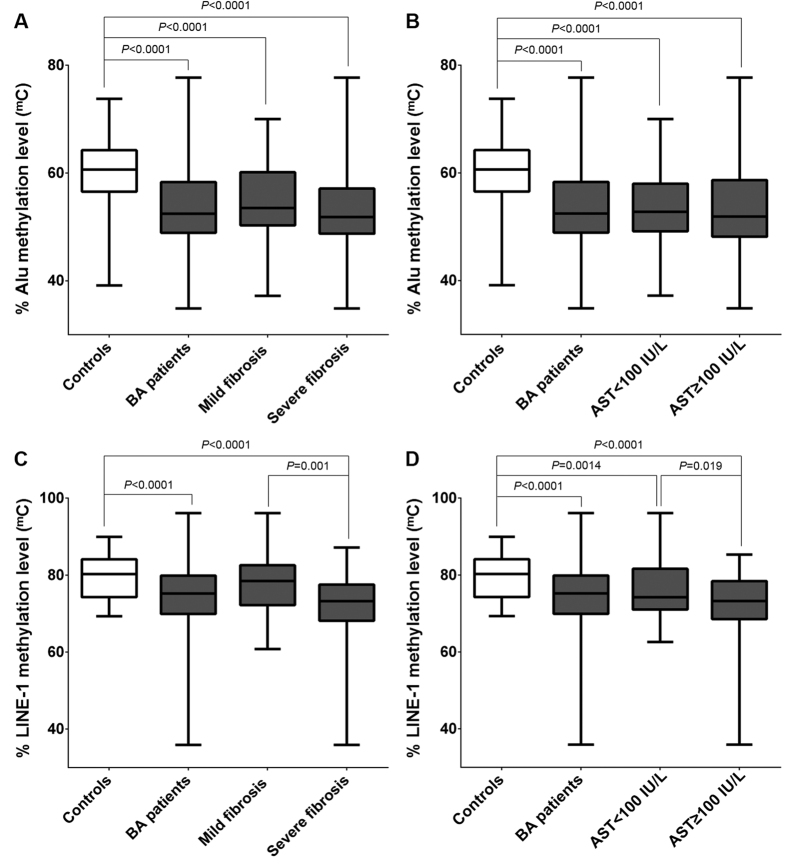
Alu and LINE-1 methylation levels among groups. (**A**) Alu methylation level in controls and BA according to fibrosis status; (**B**) severity of hepatic injury; (**C**) LINE-1 methylation level in control and BA according to fibrosis status; (**D**) severity of hepatic injury.

When disease severity was considered, BA patients were classified according to liver fibrosis status and hepatic dysfunction marker (AST value). Alu methylation levels in the different subgroups were remarkably lower than in controls (*P* < 0.0001); however, there were no significant differences in Alu methylation between early-stage (mild fibrosis and low AST value) and late-stage (severe fibrosis and high ATS value) BA patients (Fig. 2A,B). Notably, LINE-1 hypomethylation was observed in advanced BA patients with severe fibrosis and high AST value, when compared with patients with early-stage disease (*P* = 0.001 and *P* = 0.019, respectively) (Fig. 2C,D).

### Alu and LINE-1 hypomethylation in monozygotic twins discordant for biliary atresia

Subsequently, we investigated Alu and LINE-1 methylation levels in two sets of monozygotic twins discordant for BA. Set 1: the patient was a nine-year-old girl who was diagnosed with BA, while her sister was born healthy and remains so to date. Expectedly, this case demonstrated slightly lower Alu methylation level than control (58.37% *vs.* 59.62%, respectively). Set 2: the patient was a nineteen-year-old woman diagnosed as BA, with a twin sister who is healthy and has normal liver function tests. We also observed a slight reduction in Alu methylation level in this case when compared to control (57.28% *vs.* 57.84%, respectively). This effect was restricted to LINE-1 methylation level comparisons.

### Association between global methylation and risk of BA

Using unconditional logistic regression models, we evaluated Alu or LINE-1 methylation levels as an independent risk factor of BA. As shown in Table 1, this study demonstrated that overall Alu and LINE-1 methylation were inversely associated with risk of BA (OR: 0.88, 95% CI: 0.84–0.92; *P* < 0.0001 and OR: 0.89, 95% CI: 0.85–0.94; *P* < 0.0001, respectively). After adjusting for age and gender, a 4.07-fold (95% CI: 2.27–7.32) higher risk of BA was observed among individuals with lower Alu methylation below the median distribution in the controls, compared with individuals with higher Alu methylation (*P* < 0.0001), consistent with LINE-1 methylation analysis (OR: 3.51, 95% CI: 1.87–6.59; *P* < 0.0001). We further evaluated a significant dose-response association between Alu or LINE-1 hypomethylation and increased BA risk. Compared with individuals in the highest Alu methylation tertile (third tertile), individuals in the lowest tertile (first tertile) were associated with a 9.98-fold increased risk of BA (*P*-trend < 0.0001). In addition, there was a significant dose-response association between the lowest LINE-1 methylation tertile and increased risk of BA (*P*-trend < 0.0001). Specifically, when using the third tertile (the highest tertile) as the reference group, adjusted ORs for the first and second tertile were 6.52 (95% CI: 2.79–15.27) and 2.83 (95% CI: 1.17–6.88), respectively.

### Correlation between global methylation and clinical parameters

To further determine possible correlations between Alu and LINE-1 methylation, as well as biochemical variables in BA patients, we performed multiple linear regression analysis with adjustments for confounding variables. Relationships between global DNA methylation and clinical outcomes are presented in Table 2. There were no statistically significant associations between Alu methylation and clinical outcomes in BA patients. In contrast, a reduction in LINE-1 methylation was found to be associated with increased liver stiffness (β coefficient = −0.17, 95% CI: −0.24 to −0.10; *P* < 0.0001).

### Increased 8-hydroxy-2′-deoxyguanosine levels

**Figure 3:**
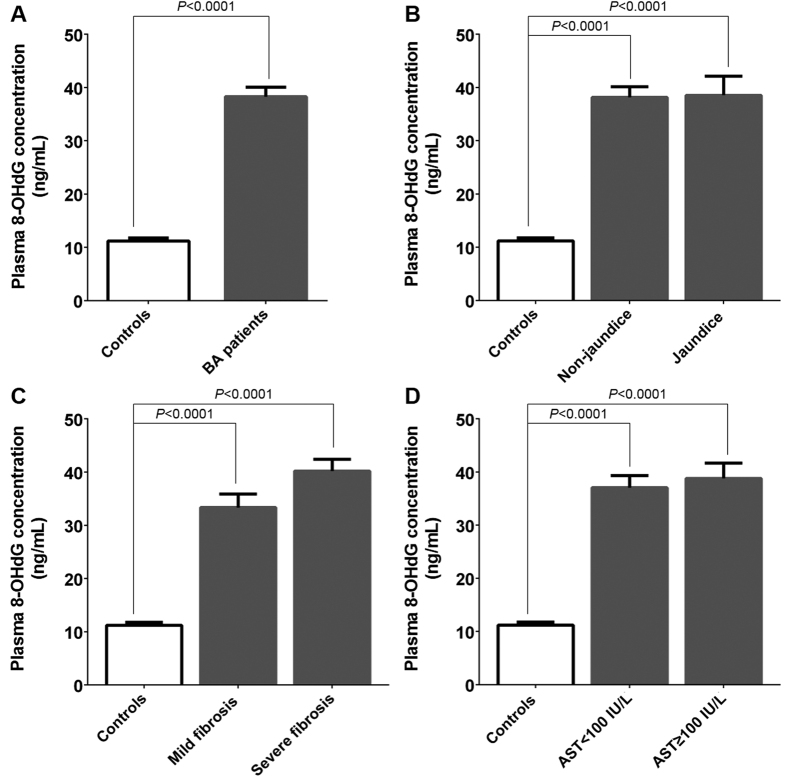
Plasma 8-hydroxy-2′-deoxyguanosine levels of subjects among groups. (**A**) plasma 8-OHdG levels in BA patients and healthy controls; (**B**) BA patients with and without jaundice; (**C**) BA subgroups, including mild fibrosis (F0-F2) and severe fibrosis (F3–F4); (**D**) early-stage or late-stage of hepatic dysfunction in BA patients based on AST value.

To assess levels of oxidative DNA damage in BA, we measured circulating 8-OHdG concentrations in 114 BA patients and 53 healthy controls. Mean plasma 8-OHdG value in BA patients was considerably higher than unaffected controls (*P* < 0.0001) (Fig. 3A). In analyses stratified by disease severity, BA patients were categorized based on jaundice status, fibrosis status, and hepatic dysfunction marker (AST value). Elevated plasma 8-OHdG concentrations was found in advanced BA patients with persistent jaundice, severe fibrosis, and advanced stage of hepatic injury, as compared to healthy controls (*P* < 0.0001, *P* < 0.0001, and *P* < 0.0001, respectively). Contrariwise, no significant differences in 8-OHdG concentrations were noted in comparison with concentrations in patients with early-stage and late-stage, as demonstrated in Fig. 3B–D.

### Relationships between global methylation, oxidative DNA damage, and telomere length

**Figure 4:**
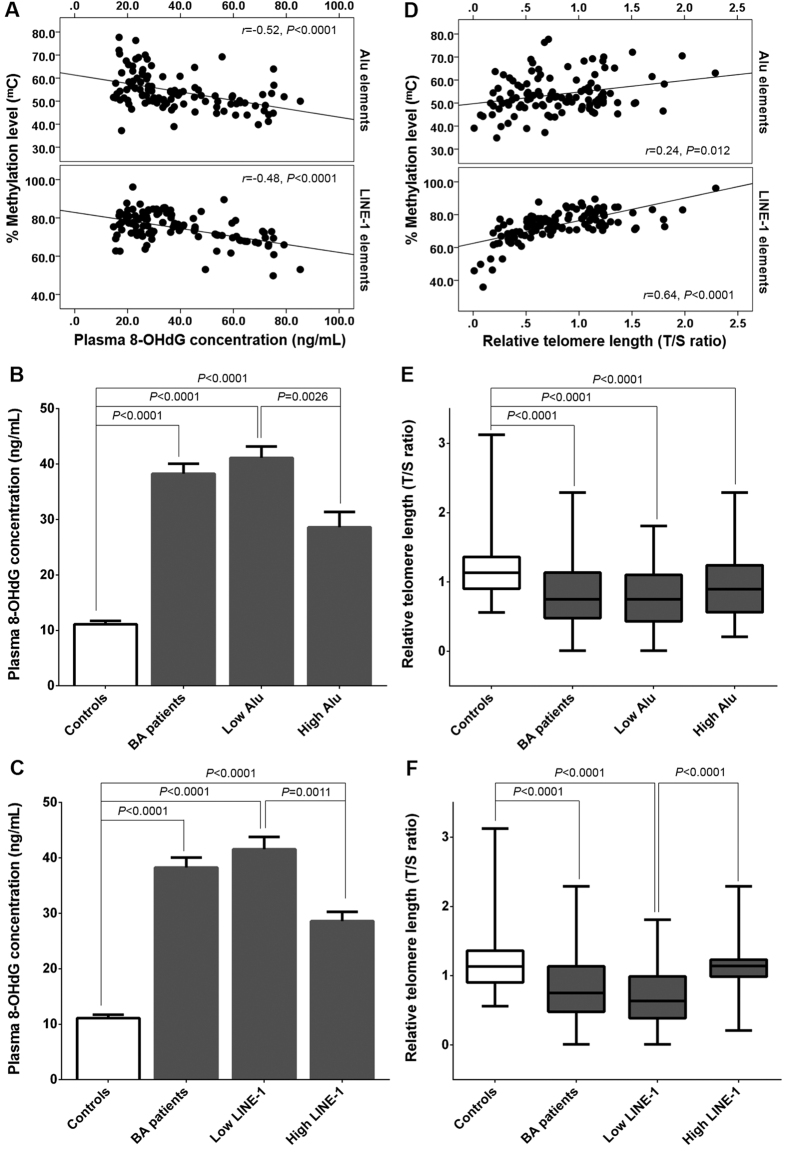
Relationships between global methylation, 8-hydroxy-2′-deoxyguanosine, and telomere length in BA. (**A**) negative correlations between Alu or LINE-1 methylation and 8-OHdG; (**B**) plasma 8-OHdG levels in BA patients with hypo- and hypermethylated status of Alu elements; (**C**) plasma 8-OHdG levels in BA patients with hypo- and hypermethylated status of LINE-1 elements; (**D**) positive associations between Alu or LINE-1 methylation and telomere length; (**E**) relative telomere length in BA patients with hypo- and hypermethylated status of Alu elements; (**F**) relative telomere length in BA patients with hypo- and hypermethylated status of LINE-1 elements.

Given that epigenetic modifications in global methylation may be involved in telomere elongation and may be induced by oxidative DNA damage, we evaluated associations between methylation of Alu or LINE-1, plasma 8-OHdG, and telomere maintenance. A significantly negative association between Alu methylation and plasma 8-OHdG was observed in BA patients (*r* = −0.52, *P* < 0.0001). Moreover, LINE-1 methylation was inversely correlated with plasma 8-OHdG in BA patients (*r* = −0.48, *P* < 0.0001), as represented in Fig. 4A. To better understand the relationship between global methylation and oxidative DNA damage, we also separately evaluated BA patients with global hypomethylation or hypermethylation. Mean plasma 8-OHdG concentrations in BA patients with Alu hypomethylation were remarkably higher than both patients with Alu hypermethylation and healthy controls (*P* = 0.0026 and *P* < 0.0001, respectively) (Fig. 4B). Patients with LINE-1 hypomethylation had consistently significantly higher plasma 8-OHdG concentrations than patients with hypermethylation of LINE-1 elements and unaffected controls (*P* = 0.0011 and *P* < 0.0001, respectively) (Fig. 4C).

We further examined correlations between changes in Alu or LINE-1 methylation and telomere length in BA patients and observed a weak positive correlation between Alu methylation and telomere length (*r* = 0.24, *P* = 0.012). Correspondingly, LINE-1 methylation levels were positively associated with telomere length (*r* = 0.64, *P* < 0.0001) (Fig. 4D). We also compared relative telomere length in BA patients with Alu or LINE-1 hypomethylation and hypermethylation. Subsequent analysis showed that BA patients with LINE-1 hypomethylation had significantly shorter telomere length than those with LINE-1 hypermethylation and healthy controls (*P* < 0.0001). This effect did not vary in comparison between patients with Alu hypomethylation and hypermethylation (Fig. 4E,F).

## Discussion

This study investigated the effectiveness of repetitive elements methylation in peripheral blood leukocytes as a proxy for global methylation in postoperative BA patients. We found that Alu and LINE-1 elements were robustly hypomethylated in BA patients, as compared to healthy controls. Reduction of both Alu and LINE-1 methylation levels was also associated with increased risk of BA. Importantly, LINE-1 methylation was associated with poor outcomes in BA patients. Moreover, Alu and LINE-1 methylation levels were significantly related with oxidative DNA damage and relative telomere length. These findings support the notion that there exists epigenetic mechanism associated with genomic instability in the pathogenesis of BA.

Methylation of retrotransposable elements has been shown to be associated with global genomic methylation. Hypomethylation in these elements may increase their activity as retrotransposon sequences, resulting in genomic alterations and more mutations by several different mechanisms^20^. To our knowledge, this is the first study to explore relationships between Alu or LINE-1 methylation, oxidative DNA damage, telomere length, and hepatic dysfunction in BA patients. Here, we report hypomethylation of both Alu and LINE-1 elements in BA patients, which was supported by decreased Alu methylation levels in two BA patients compared to those in their respective monozygotic twin sisters. In accord with our findings, Alu hypomethylation has been observed in post-menopausal women with osteoporosis^10^ and patients with glioma cancer^11^. Furthermore, LINE-1 methylation has been reported in hepatocellular carcinoma patients^12^,^13^. It is well known that LINE-1 elements encode enzymes that allow them to replicate and insert themselves into different genomic regions, altering transcription and translation into functional proteins^21^,^22^. Transcription of LINE-1 elements has been shown to contribute to transcriptional regulation of human development genes and cell differentiation^23^,^24^. Our observation regarding association of LINE-1 methylation with poor outcome in BA patients supports prior evidence that epigenetic modifications play important roles in BA etiology^25^.

The current study showed that plasma 8-OHdG concentrations were significantly higher in BA patients than in controls. Consistent with this finding, previous study demonstrated that 8-OHdG, oxidative stress marker, was highly expressed in liver tissues of BA patients^26^. Tiao *et al.* also reported that hepatic 8-OHdG expression in early-stage BA patients was substantially greater than in patients with choledochal cyst^27^. Subsequent analysis revealed elevation of plasma 8-OHdG in BA patients with both Alu and LINE-1 hypomethylation. Furthermore, Alu and LINE-1 methylation levels were inversely correlated with plasma 8-OHdG levels in BA patients.

Previous investigation has documented the role of global DNA methylation in the variability of telomere length^28^. Telomeres are repeated DNA sequences of TTAGGG and an associated protein complex at chromosome ends that are essential for maintaining chromosome integrity^29^. With each cell division, telomeres shorten due to the inability of DNA polymerases to replicate the ends of linear molecules and also due to nucleolytic degradation, oxidative DNA damage, and inflammation^30^. Our recent study has provided evidence for telomere shortening in age-associated biliary atresia^31^; however, this causal relation remains largely unknown. Epigenetic mechanism also appears to be an important component of telomere length regulation. Importantly, DNA hypomethylation, especially in subtelomeric DNA repeats, was associated with telomere shortening that may result from mutation in the DNA methyltransferase 3b gene^32^, suggesting a regulatory role of DNA methylation on telomere length. In this study, we showed positive correlations between Alu and LINE-1 methylation with telomere length in BA patients. In agreement with these findings, LINE-1 methylation was positively associated with telomere length in dyskeratosis congenital^33^. Wong *et al.* recently reported positive relationships between both Alu and LINE-1 methylation levels and telomere length^34^. Notably, we found that BA patients with LINE-1 hypomethylation had significantly shorter telomere length than those with LINE-1 hypermethylation. Given their sequence contexts, LINE-1 elements comprise a greater number of bases in subtelomeric regions across the genome than do Alu elements^35^.

The limitation of this study should be considered. First, measurement of global methylation was performed with DNA from peripheral blood leukocytes, which may not reflect methylation levels in tissue-specific liver cells; however, global methylation in leukocyte DNA has been shown to be associated with BA development^36^. Second, white blood cell differentials were not measured in the present study. Peripheral blood leukocytes contain a heterogeneous mixture of cell types, each cell population contributing its own unique methylation and telomere length to the final analysis. Therefore, further studies on differential analyses of white blood cells will be necessary in order to validate that apparent differences in global methylation and/or telomere length are not in fact differences in leukocyte cell type composition. Additionally, because the subjects in this study are from hospital-based participants rather than the general population, there might be some risk of selection bias if they had any differences in terms of the studied exposures. Moreover, the timing of blood draws varied with respect to time since diagnosis and treatment, which introduces uncertainty regarding correlations between clinical outcomes and Alu hypomethylation. Thus, the associations identified in leukocyte DNA may represent either causal, consequential or coincidental relationships. Longitudinal or prospective cohort studies will be needed to verify the risk-effect of global hypomethylation on BA susceptibility. Furthermore, DNA methylation level estimations may be confounded by other factors such as environmental exposures, parental smoking, socioeconomic status, ethnicity, body mass index, and lifestyle habits. Unfortunately, such information would be unavailable due to limitations of records accessibility. Therefore, residual confounding might still exist. To address these challenges, future studies should collect prospective measurements of these data to preclude bias and reverse causation. Lastly, sample size of BA subgroups was relatively small. This factor diminished the power of statistics, resulting in a failure to observe significant differences of Alu methylation among BA subgroups. Larger studies with various ethnic groups/races are warranted to evaluate the differences between subgroups.

To sum up, this study reported that, independent of risk factors, hypomethylation of retrotransposable DNA elements in peripheral blood leukocytes was associated with shorter telomeres, elevated oxidative DNA damage, and a higher risk of BA. Accordingly, hypomethylation of retrotransposable DNA elements in peripheral blood leukocytes may serve as a potential biomarker for BA susceptibility. Examinations to elucidate whether genome-wide methylation in peripheral blood reflects epigenetic changes in liver tissue will be essential to elicit and identify the role of epigenetics in BA. Future research in both gene-specific methylation and potential underlying mechanisms related to retrotransposon methylation will help to elucidate the effect of epigenetic alterations in BA etiology, potentially yielding new diagnostic and therapeutic approaches in BA.

## Methods

### Study participants

The study protocol conformed to the ethical standards outlined in the Declaration of Helsinki and was approved by the Institutional Review Board (IRB) of the Faculty of Medicine, Chulalongkorn University. All participants, parents, or legal guardians were fully informed regarding the study protocol and procedures prior to participating in the study. Written informed consent was obtained from all patients and from parents or legal guardians of patients younger than 18 years of age.

This case-control study consisted of 114 BA patients and 114 age-matched unaffected volunteers with no underlying liver disease. All BA patients were diagnosed by intraoperative cholangiography and were surgically treated with Kasai portoenterostomy. Healthy controls who participated in an evaluation of hepatitis B vaccine and attended the Well Baby Clinic at King Chulalongkorn Memorial Hospital for vaccination had normal physical findings and no underlying disease. In addition, two pairs of monozygotic girl twins with BA discordance were enrolled in this study. We classified BA patients according to serum total bilirubin (TB) into either the non-jaundice group (TB < 2 mg/dL; n = 77) or the persistent jaundice group (TB ≥ 2 mg/dL; n = 37). BA patients were stratified according to severity of liver fibrosis into either the mild fibrosis group (F0-F2: 0-9.7 kPa; n = 32) or the severe fibrosis group (F3-F4: > 9.7 kPa; n = 82). Based on severity of hepatic dysfunction [aspartate aminotransferase (AST) value], BA patients were also categorized as either early-stage (AST < 100 IU/L; n = 56) or late-stage (AST ≥ 100 IU/L; n = 58).

Blood samples from participants were collected in ethylenediaminetetraacetic acid (EDTA) tubes to facilitate isolation of plasma and leukocytes and were then stored at −80 °C until analysis.

### Clinical assessments of outcomes

All liver function analyses, including TB, AST, alanine aminotransferase (ALT), alkaline phosphatase (ALP), and albumin were performed on a Roche Hitachi 912 chemistry analyzer (Roche Diagnostics, Basel, Switzerland). Measurement of liver stiffness by transient elastography was performed using a Fibroscan (EchoSens, Paris, France). Briefly, assessments were performed by placing a Fibroscan transducer probe on the intercostal space at the area of the right lobe of the liver. Measurements were then performed until 10 validated results were obtained with a success rate of at least 80%. The median value of 10 validated scores represented the elastic modulus measurement of the liver, which was expressed in kilopascals (kPa).

### Alu and LINE-1 methylation analysis

Genomic DNA was extracted from peripheral blood leukocytes using GE Healthcare DNA Purification Kit (Buckinghamshire, UK). Extracted DNA (50 ng; concentration: 2.5 ng/μL) was treated by EZ DNA Methylation Gold Kit (Zymo Research, Orange, CA, USA), according to manufacturer’s protocol.

DNA methylation was quantitated by qCOBRA using previously described primers and conditions^37^. Primers used for COBRA Alu and COBRA LINE-1 amplifications were, as follows: Alu forward primer 5′-GGRGRGGTGGTTTARGTTTGTAA-3′; Alu reverse primer 5′-CTAACTTTTTATATTTTTAATAAAAACRAAATTTCACCA-3′; LINE-1 forward primer 5′-GTTAAAGAAAGGGGTGAYGGT-3; and, LINE-1 reverse primer 5′-AATACRCCRTTTCTTAAACCRATCTA-3′. Both PCRs were functioned in a final volume of 10 μL, containing 2.5 ng of bisulfite-treated DNA, 10X PCR buffer, 25 mM MgCl_2_, 200 mM dNTPs, 20 μM primers, and 0.5 U Taq DNA polymerase (HotStar, Qiagen, Valencia, CA, USA). PCR cycling conditions started with a 95 °C incubation for 15 min, followed by 40 cycles of 95 °C for 45 sec, then 57 °C (for Alu) or 55 °C (for LINE-1) for 45 sec and 72 °C for 45 sec, and finally 72 °C for 7 min. After PCR amplification, Alu amplicons (133 bp) were subsequently digested with 2 U *TaqI* in *TaqI* buffer (MBI Fermentas, Burlington, Canada), while LINE-1 amplicons (92 bp) were digested with 2 U *TaqI* and 8 U *TasI* in NEBuffer 3 (New England Biolabs, Ontario, Canada). Both digestion reactions were incubated at 65 °C overnight, followed by separation on an 8% non-denaturing polyacrylamide gel. Gels were then stained with ethidium bromide and band intensities were analyzed by Molecular Imager Gel Doc using Image Lab Software (Bio-Rad, Begoniastraat, Belgium).

Both qCOBRA Alu and qCOBRA LINE-1 were stratified into four patterns depending on methylation status of two CpG dinucleotides, as follows: hypermethylation (^m^C^m^C), partial methylation (^m^C^u^C and ^u^C^m^C), and hypomethylation (^u^C^u^C). Methylation levels and patterns of both Alu and LINE-1 were measured to determine the precise percentage of methylated CpG dinucleotides. For Alu methylation analysis, we measured the percentage of Alu methylation levels and patterns in each group based on the intensity of the COBRA-digested Alu products. DNA fragments derived from enzymatic digestion of COBRA-Alu products were divided into six fragments of 133, 90, 75, 58, 43, and 32 bp, which represented different methylation states. Percentage of each methylation pattern was estimated, as follows: A = intensity of the 133 bp fragment divided by 133; B = intensity of the 58 bp fragment divided by 58; C = intensity of the 75 bp fragment divided by 75; D = intensity of the 90 bp fragment divided by 90; E = intensity of the 43 bp fragment divided by 43; and, F = intensity of the 32 bp fragment divided by 32. The percentage of each Alu element methylation pattern was then calculated, as follows: percentage of Alu methylation level (%^m^C) = 100 × (E + B)/(2 A + E + B + C + D); percentage of hypermethylated loci (%^m^C^m^C) = 100 × F/(A + C + D + F); percentage of both partially methylated loci (%^u^C^m^C) = 100 × C/(A + C + D + F); (%^m^C^u^C) = 100 × D/(A + C + D + F); and, percentage of hypomethylated loci (%^u^C^u^C) = 100 × A/(A + C + D + F).

For LINE-1 methylation analysis, DNA fragments from enzymatic digestion for qCOBRA LINE-1 were separated into five fragments: 92 bp, 60 bp, 50 bp, 42 bp, and 32 bp. The number of CpG dinucleotides was determined by dividing each band intensity by the length (bp) of the double-stranded DNA fragment, as follows: A = 92 bp fragment intensity/92; B = 60 bp fragment intensity/56; C = 50 bp fragment intensity/48; D = 42 bp fragment intensity/40; E = 32 bp fragment intensity/28; and, F = [(D + E) − (B − C)]/2. LINE-1 methylation levels were calculated using the number of CpG dinucleotides according to the following formulas: LINE-1 methylation level percentage (%^m^C) = 100 × (A + 2C + F)/(2A + 2B + 2C + 2F); hypermethylated loci percentage (%^m^C^m^C) = 100 × (C/2)/[(C/2) + A + B + F]; both of partially methylated loci percentage (%^u^C^m^C) = 100 × F/[(C/2) + A + B + F); (%^m^C^u^C) = 100 × A/[(C/2) + A + B + F); and, hypomethylated loci percentage (%^u^C^u^C) = 100 × B/[(C/2) + A + B + F]. DNA samples from HeLa, Jurkat, and Daudi cell lines were used as positive controls to normalize inter-assay variations in all experiments.

### Quantitation of 8-hydroxy-2′deoxyguanosine

Plasma 8-OHdG levels were quantitatively determined from venous blood samples using a commercial sandwich enzyme-linked immunosorbent assay (ELISA) kit (Trevigen, Gaithersburg, MD, USA), according to manufacturer’s instructions. Antibodies specific to 8-OHdG generated by the entire immunogen were utilized. Twofold serial dilutions of 8-OHdG standard with a concentration of 0.89–56.7 ng/mL were used as standards. Intra-assay and inter-assay precision were less than 10% and 15%, respectively. The sensitivity of this assay was 0.57 ng/mL.

### Telomere length measurement

Telomere length in genomic DNA was estimated by applying a quantitative real-time polymerase chain reaction (PCR) method originally described by Cawthon^38^. Briefly, PCRs were performed using StepOnePlus™ Real-Time PCR System (Applied Biosystems, Foster City, CA, USA) with SYBR Green fluorescence (RBC Bioscience, Taipei, Taiwan). Relative telomere length was measured according to the ratio of the telomere repeat copy number (T) to the single-copy gene copy number (S) in each given sample. In each sample, the quantity of telomere repeats and the quantity of single-copy genes were normalized to a reference DNA sample (from a single individual).

### Statistical analysis

All statistical analyses were performed using SPSS Statistics version 22.0 (SPSS, Inc., Chicago, IL, USA). Statistical significance between clinical parameters of healthy controls and BA groups was determined by Student’s *t*-test. Kolmogorov-Smirnov test and quantile-quantile (q-q) plot were used to evaluate Alu and LINE-1 methylation levels for normal distribution. Given that Alu and LINE-1 methylation levels were found not to be normally distributed, significance of changes in these methylations was calculated by Mann-Whitney *U* test or Kruskal-Wallis H test for continuous variables. Unconditional logistic regression models were used to estimate associations between methylation of Alu or LINE-1 and BA risk using odds ratio (OR) and 95% confidence interval (CI), with adjustments for confounding factors including age and gender. We used linear regression models to evaluate potential predictors of Alu or LINE-1 methylation levels as continuous variables. Spearman’s rank correlation coefficient test was used to estimate relationships between global methylation, telomere length, and circulating 8-OHdG levels. Data were expressed as mean ± standard error of the mean (SEM). All statistical tests were based on two-tailed probability, with *P*-values less than 0.05 considered statistically significant.

## Additional Information

**How to cite this article**: Udomsinprasert, W. *et al.* Global methylation, oxidative stress, and relative telomere length in biliary atresia patients. *Sci. Rep.* **6**, 26969; doi: 10.1038/srep26969 (2016).

## Supplementary Material

Supplementary Information

## Figures and Tables

**Table 1:** Association between global methylation and risk of BA.

	BA	Controls	Unadjusted OR	*P*-value	Adjusted^a^ OR	*P*-value
			OR (95% CI)		OR (95% CI)	
Alu elements						
Overall	100.00%	100.00%	0.88 (0.84–0.97)	< 0.001	0.88 (0.84–0.92)	< 0.0001
By median						
Low	78.07%	50.00%	4.07 (2.27–7.33)	< 0.0001	4.07 (2.27–7.32)	< 0.0001
High	21.93%	50.00%	1.00 (reference)		1.00 (reference)	
By tertile						
1^st^ tertile	73.68%	33.33%	9.95 (4.54–21.80)	< 0.0001	9.98 (4.55–21.89)	< 0.0001
2^nd^ tertile	14.46%	33.33%	2.53 (1.03–6.20)	0.04	2.51 (1.02–6.16)	0.04
3^rd^ tertile	12.28%	33.33%	1.00 (reference)		1.00 (reference)	
*P*-trend				< 0.0001		< 0.0001
LINE-1 elements						
Overall	100.00%	100.00%	0.90 (0.85–0.94)	< 0.0001	0.89 (0.85–0.94)	< 0.0001
By median						
Low	77.19%	50.00%	3.53 (1.88–6.61)	< 0.0001	3.51 (1.87–6.59)	< 0.0001
High	22.81%	50.00%	1.00 (reference)		1.00 (reference)	
By tertile						
1^st^ tertile	62.28%	33.33%	6.46 (2.78–15.00)	< 0.0001	6.52 (2.79–15.27)	< 0.0001
2^nd^ tertile	21.93%	33.33%	2.82 (1.17–6.82)	0.02	2.83 (1.17–6.88)	0.02
3^rd^ tertile	15.79%	33.33%	1.00 (reference)		1.00 (reference)	
*P-*trend				< 0.0001		< 0.0001

^a^Unconditional logistic regression analysis, adjusted for age and gender; *P*-value < 0.05 indicates statistical significance.

**Table 2:** Multivariate linear regression analysis of global methylation estimates.

Variables	Alu methylation^a^		LINE-1 methylation^a^	
	β coefficients (95% CI)	*P*-value	β coefficients (95% CI)	*P*-value
Age (years)	−0.12 (−0.49 to 0.25)	0.52	−0.14 (−0.51 to 0.24)	0.50
Gender	−1.47 (−4.72 to 1.79)	0.37	2.40 (−1.10 to 5.78)	0.16
Liver stiffness (kPa)	0.03 (−0.04 to 0.10)	0.38	−0.17 (−0.24 to −0.10)	< 0.0001
TB (mg/dL)	−0.14 (−0.69 to 0.42)	0.63	0.27 (−0.29 to 0.84)	0.34
AST (IU/L)	0.00 (−0.03 to 0.04)	0.96	0.02 (−0.02 to 0.07)	0.30
ALT (IU/L)	0.00 (−0.03 to 0.04)	0.85	−0.01 (−0.05 to 0.02)	0.46
ALP (IU/L)	0.00 (−0.01 to 0.01)	0.99	0.00 (−0.01 to 0.01)	0.76
Albumin (g/dL)	1.03 (−0.80 to 2.86)	0.27	−1.13 (−3.02 to 0.77)	0.24

^a^Unconditional logistic regression analysis, adjusted for age, gender, liver stiffness, total bilirubin (TB), aspartate aminotransferase (AST), alanine aminotransferase (ALT), alkaline phosphatase (ALP), and albumin; *P*-value < 0.05 indicates statistical significance.
