# Supplementary material for: Global methylation, oxidative stress, and relative telomere length in biliary atresia patients
Source: Sci Rep. 2016 May 31;6:26969. doi: 10.1038/srep26969 (PMC4886632; doi:10.1038/srep26969)
Supplement: Supplementary Information [file srep26969-s1.pdf]

Global methylation, oxidative stress, and relative telomere length in biliary atresia patients

Wanvisa Udomsinprasert[1], Nakarin Kitkumthorn[2], Apiwat Mutirangura[3], Voranush Chongsrisawat[4], Yong Poovorawan[4], Sittisak Honsawek[1]

Supplementary Table 1. Demographic and clinical characteristics of study participants.

**Characteristics**	**BA patients (n=114)**	**Healthy controls (n=114)**	***P*-value**
**Age (years)**	8.95±0.45	8.95±0.45	1.00
**Gender (female:male)**	66:48 (57.89%:42.11%)	64:50 (56.14%:43.86%)	0.79
**Liver stiffness (kPa)**	32.78±2.38	4.01±0.19	<0.0001
**TB (mg/dL)**	2.72±0.37	-	NA
**AST (IU/L)**	117.92±9.27	26.66±0.82	<0.0001
**ALT (IU/L)**	97.23±8.38	9.24±0.65	<0.0001
**ALP (IU/L)**	368.17±27.61	-	NA
**Albumin (mg/dL)**	4.24±0.10	-	NA

Data presented as mean ± standard error of the mean, unless otherwise specified;

P-value<0.05 indicates statistical significance.

Abbreviations: BA, biliary atresia; TB, total bilirubin; AST, aspartate aminotransferase; ALT, alanine aminotransferase; ALP, alkaline phosphatase; NA, not available.
